# Supplementary material for: Single domain spectroscopic signatures of a magnetic kagome metal
Source: Nat Commun. 2026 Apr 17;17:3571. doi: 10.1038/s41467-026-71924-9 (PMC13090345; doi:10.1038/s41467-026-71924-9)
Supplement: Supplementary file 1 — Supplementary Information [file 41467_2026_71924_MOESM1_ESM.pdf]

SUPPLEMENTARY MATERIAL: Single domain spectroscopic signatures of a magnetic Kagome metal

L. Plucinski,[1,2,*] G. Bihlmayer,[3] Y. Mokrousov,[3,4] Yishui Zhou,[5] Yixi Su,[5] J. D. Denlinger,[6] A. Bostwick,[6] C. Jozwiak,[6] E. Rotenberg,[6] D. Usachov,[7] and C. M. Schneider[1,8,9]

[1]*Peter Grünberg Institut (PGI-6), Forschungszentrum Jülich GmbH, 52428 Jülich, Germany*

[2]*Institute for Experimental Physics II B, RWTH Aachen University, 52074 Aachen, Germany*

[3]*Peter Grünberg Institut (PGI-1), Forschungszentrum Jülich and JARA, 52428 Jülich, Germany*

[4]*Institute of Physics, Johannes-Gutenberg University Mainz, 55099 Mainz, Germany*

[5]*Jülich Centre for Neutron Science (JCNS) at Heinz Maier-Leibnitz Zentrum (MLZ), Forschungszentrum Jülich, Lichtenbergstrasse 1, D-85747 Garching, Germany*

[6]*Advanced Light Source, Lawrence Berkeley National Laboratory, One Cyclotron Road, Berkeley, CA 94720, USA*

[7]*Donostia International Physics Center (DIPC), 20018 Donostia-San Sebastian, Spain*

[8]*Fakultät für Physik, Universität Duisburg-Essen, 47048 Duisburg, Germany*

[9]*Physics Department, University of California, Davis, CA 95616, USA*

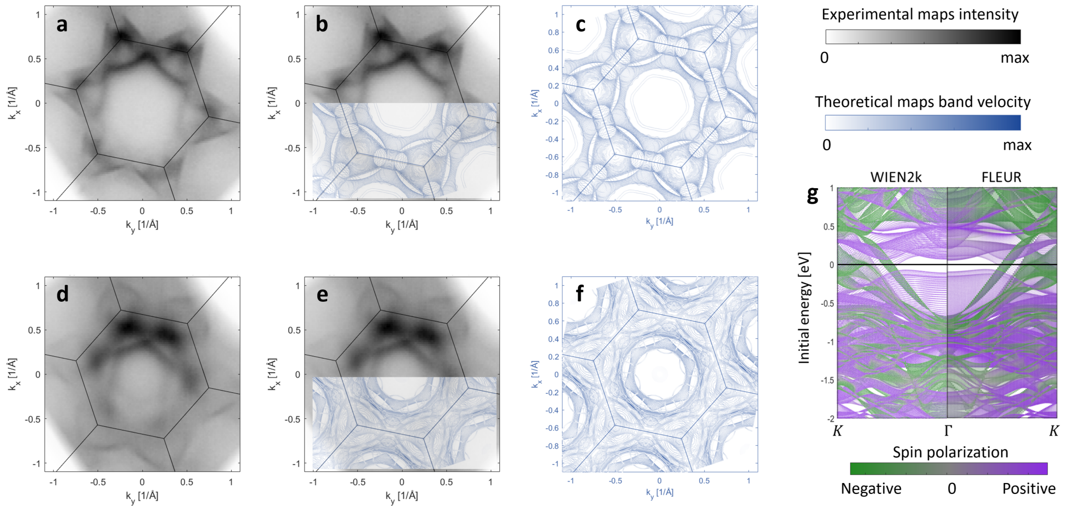

FIG. S1. Experimental and calculated constant-energy maps of the valence electronic structure. **a-c** Experimental and theoretical constant energy maps at the Fermi level at $h\nu = 140$ eV. **d-f** Experimental map at 190 meV compared to the theoretical map at 250 meV. Solid lines in **a-f** represent surface Brillouin zones. **g** Comparison between projected band structure calculated using WIEN2k [1] and FLEUR [2]. Theory maps in **b,c,e,f** were calculated using WIEN2k.

SI. EXPERIMENTAL AND THEORETICAL BAND DISPERSIONS

Figure S1 shows a comparison between experimental and theoretical constant energy maps (CEMs) at $h\nu = 140$ eV and two binding energies. In the theoretical CEMs we highlighted highly dispersing bands, since these bands are appear intense in the experimental maps (see Fig. 4 of the main text).

One can see that most of the observed experimental features can be interpreted as stemming from the bulk projected bands. This concerns both the electron pocket centered at Γ and the shape of the features around K points.

Theory CEMs were calculated using WIEN2k [1] with the 51×51 k-point mesh in the surface Brillouin zone. In Fig. S1**g** we show that bands calculated using WIEN2k and FLEUR [2] are in good agreement.

These results motivate the interpretation of the experimental band dispersions as being predominantly due to projected bulk bands.

* l.plucinski@fz-juelich.de

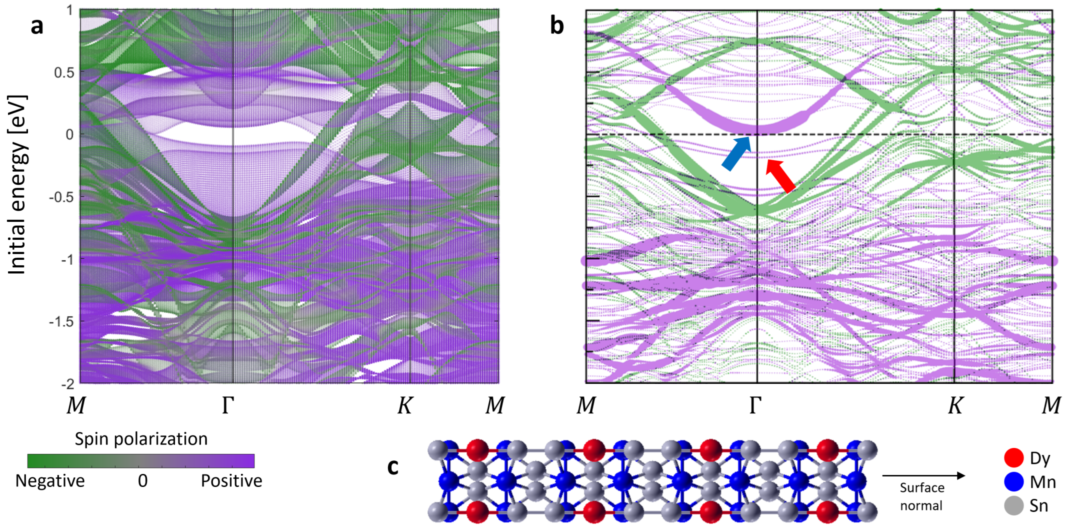

FIG. S2. Comparison between theoretical bulk and surface electronic structure, both calculated using FLEUR [2]. **a** Projected bulk band structure. **b** Surface electronic structure calculated using the slab geometry shown in **c**. False color in **a,b** indicates spin polarization.

SII. SURFACE ELECTRONIC STRUCTURE

Figure S2 shows a comparison between the projected band structure and surface electronic structure performed in the periodic slab geometry. The surface electronic structure is in good agreement to the previous calculations [3]. On the other hand, the thickness of the slab is not sufficient, which is exemplified by sparse bands in Fig. S2**b** as indicated by the red arrow. The corresponding energy-momentum region in the bulk band structure in S2**a** is filled with projected bands. Therefore, the bands indicated by red arrow are not surface bands.

On the other hand, the band indicated by blue arrow in Fig. S2**b** likely represents a surface-localized band, however, its position might be influenced by the insufficient slab thickness.

There also exist further features in S2**b** that might indicate surface states. However, in order to provide further detailed interpretation of these features a calculation for a larger slab should be performed, which is computationally expensive.

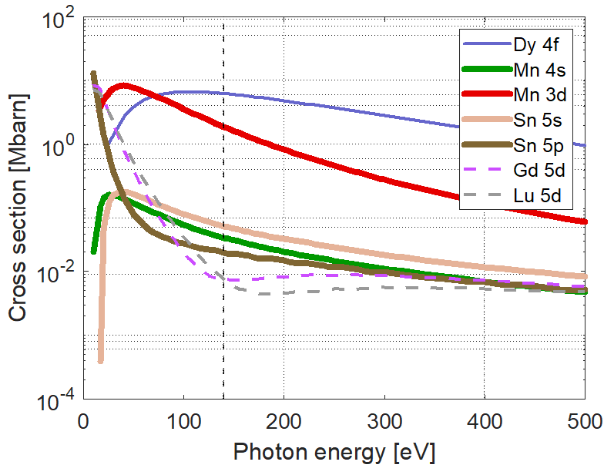

FIG. S3. Atomic cross sections for relevant valence band orbitals. Dashed vertical line indicates $h\nu = 140$ eV at which majority of our experiments have been performed. Dy $4f$ is also shown for comparison, although it does not contribute to the bands close to the Fermi level. See text for details.

SIII. PHOTOEMISSION CROSS SECTIONS

In one-electron picture, each momentum-resolved eigenvalue is connected to the real-space wave function $\psi_{\mathbf{k}}(E)$. These wave functions reflect the shapes of atomic orbitals, in particular close to the nuclei, where much of the charge is located. This allows to decompose band characters into orbital characters. Therefore, in an approximate picture, it is expected that intensities of the bands observed in ARPES follow their $h\nu$-dependent atomic cross section rules, tabulated by Yeh and Lindau [4] and available online at:

https://vuo.elettra.eu/services/elements/WebElements.html

In Fig. S3 we plot the cross sections that are relevant to the valence band region of $DyMn_6Sn_6$. One can see that Mn $3d$ strongly dominates for the $h\nu$ used in our experiments, at $h\nu = 140$ eV it is over 35 times larger than the second strongest Sn $5s$. Therefore, we expect it dominates our valence band ARPES spectra (Fig. 4 of the main text).

The Dy $5d$ is unoccupied in the atom and the cross section is not available in the tables, therefore we plot the Gd $5d$ and Lu $5d$ cross sections instead, both being similar and over 100 times smaller than Mn $3d$ for our photon energy range.

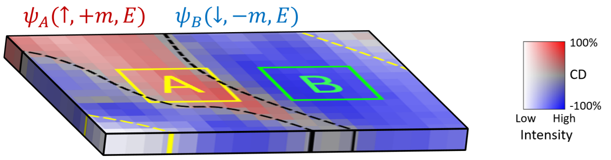

FIG. S4. Illustration of domains A and B related by time-reversal symmetry. The two domains are characterized by opposite spin and orbital configurations ($\pm m$), forming the basis for the symmetry relations used in the circular dichroism analysis discussed in Section SIV.

SIV. CIRCULAR DICHROISM FROM THE TWO ANTI-ALIGNED MAGNETIC DOMAINS

To shed some light on the question of which properties of the material are visualized by the plots in Fig. 4**c-f** of the main text, we develop here a simple model of the photoemission process using circularly polarized light. The photoemission matrix element for $C_\pm$ light can be written as

$$M_\pm = \langle \psi_f | (\varepsilon_{x'} \pm i\varepsilon_{y'}) \cdot \mathbf{p} | \psi_i \rangle = \langle \psi_f | e_\pm \cdot \mathbf{p} | \psi_i \rangle,$$

where in the coordinate system (x', y', z') the z' axis is along the incident light and should be used as a quantization axis for the determination of OAM, m_l. The matrix elements from the two domains A and B, related by time-reversal symmetry (Fig. S4), for a particular energy and crystal momentum $\mathbf{k}$ can be written as

$$M_{\pm,A} = \langle \psi_f | e_\pm \cdot \mathbf{p} | \psi_{\mathbf{k},m_l,\uparrow} \rangle$$

$$M_{\pm,B} = \langle \psi_f | e_\pm \cdot \mathbf{p} | \psi_{-\mathbf{k},-m_l,\downarrow} \rangle.$$

In first approximation we consider a central potential as scatterer that leads to initial state orbitals Y_l^m quantized along z' and ignore multiple scattering events. Imagine a mirror plane $\mathcal{M}$ spread by the potential center, light incidence and outcoming electron $\mathbf{k}_f$. Such mirror plane leads to

$$\mathcal{M}C_+ = C_-$$

$$\mathcal{M}\mathbf{k}_f = \mathbf{k}_f$$

$$\mathcal{M}Y_{l,\uparrow}^m = (-1)^m \; Y_{l,\downarrow}^{-m}.$$

Note, that we assumed that the final state is spin degenerate. Then,

$$M_{+,\uparrow} = \langle \mathbf{k}_f | e_+ \cdot \mathbf{p} | Y_{l,\uparrow}^m \rangle = \langle \mathbf{k}_f | \mathcal{M}^\dagger \mathcal{M} \; (e_+ \cdot \mathbf{p}) \; \mathcal{M}^\dagger \mathcal{M} | Y_{l,\uparrow}^m \rangle = \langle \mathcal{M}\mathbf{k}_f | \mathcal{M} \; (e_+ \cdot \mathbf{p}) \; \mathcal{M}^\dagger | \mathcal{M} Y_{l,\uparrow}^m \rangle \, .$$

Applying the mirror operations, we get

$$M_{+,\uparrow} = \langle \mathbf{k}_f | e_- \cdot \mathbf{p} | (-1)^m Y^m_{l,\downarrow} \rangle = (-1)^m M_{-,\downarrow}.$$

Therefore, for the central potential

$$|M_{\pm,\uparrow}| = |M_{\mp,\downarrow}|,$$

that is, reversed magnetization leads to the same intensity difference as reversing the light chirality, that leads to CD. This qualitatively explains the origin the intensity differences in Fig. 4**c**,**d** as well as Fig. 4**e**,**f** of the main text, but not their different appearance.

In our case, the magnetization **M** is at 45° with respect to the normal, therefore, most of the crystal symmetries are broken and the above considerations are not valid anymore. Moreover, atomic-like scattering waves exhibit multiple scattering. Figure 4**c**,**d** is dominated by these multiple scattering effects (the Daimon effect [5]) which obscures any magnetic signatures. For simplicity, one can imagine a spin-polarized spherical wave emitted from one of the Y^m_l orbitals at the Mn site and being scattered by all the other sites. This wave will exhibit angle-dependent scattering profiles due to scattering on the potentials of the neighboring sites (leading primarily, but not only, to forward scattering). Since $C_\pm$ circular light is used for excitation, the spherical wave emitted from the Y^m_l orbital at the Mn site will in general be endowed by $m \pm 1$ orbital moment through the dipole selection rules. These $m \pm 1$ spherical waves will scatter differently on neighboring atoms.

Referring to Fig. 4**c**,**d** of the main text, where we stay on the same domain and change the light helicity (from C_+ to C_-). Starting with $m = 0$ initial orbitals, the final states exhibit $m \pm 1$ for $C_\pm$, leading to a strong Daimon effect (of course, in case of the initial Y^0_2 orbital both $l \pm 1$ channels are contributing). Since most states carry $m \approx 0$, most of the signal comes from this effect and is not related to the orbital momentum of the initial state.

The situation is different in case of Fig. 4**e**,**f** of the main text. Here, the emission is from the $\pm m$ orbitals for the $\uparrow$ and $\downarrow$ domains and (in panel **e**) $M_{+,\uparrow} - M_{+,\downarrow}$ is shown. Let us again first examine the case of $m = 0$ orbitals such as Y^0_0 or Y^0_2. Here, the initial state orbital, final state orbital, and the scattering influence is the same for the $\uparrow$ and $\downarrow$ domain, and therefore the Daimon-like scattering [5] vanishes if the difference between the two domains is taken.

Moving to $m \neq 0$ orbitals, we examine the exemplary case of the $Y^{\pm 2}_2$ initial orbital and C_+ light, where for our case $m = +2$ and -2 correspond to $\uparrow$ and $\downarrow$ "domains" respectively (we are imagining an orbital embedded in the domain). The only dipole-allowed final orbital for $\uparrow$ domain is $Y^{2+1}_{2+1} = Y^3_3$. For $\downarrow$ domain there are two allowed final orbitals $Y^{-2+1}_{2+1} = Y^{-1}_3$ and $Y^{-2+1}_{2-1} = Y^{-1}_1$. Therefore, in this case, Fig. 4**e**,**f** of the main text would represent the

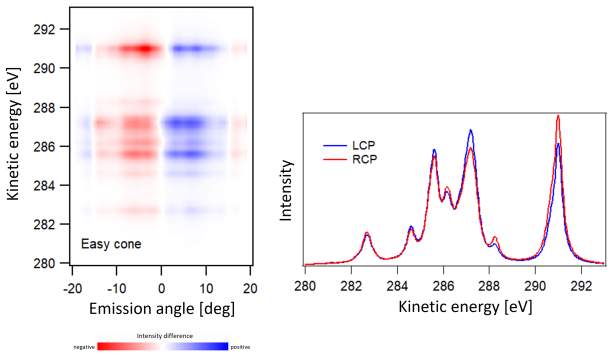

FIG. S5. Theoretical prediction for the approximate "easy cone" arrangement of **M**, sum of 12 azimuthal orientations of **M** (along all ΓM and ΓK directions), and at polar angle of $\theta = 45°$. Left: Same as Fig. 2**f** of the main text but for *easy cone*. Right: Same as Fig. 2**i** of the main text but for *easy cone*.

difference of the Y_3^3 and $\{Y_3^{-1}, Y_1^{-1}\}$ photoionization profiles. Such difference must include proper coefficients and phase shifts [6] and will exhibit a kinetic energy-dependent angular profile. Nevertheless, since the ionization profiles for the two "domains" (represented by $Y_2^{\pm 2}$) are different, they will be differently multiply scattered, potentially leading to an additional difference signal similar to the Daimon effect.

The above arguments qualitatively explain why in Fig. 4**c**,**d** the Daimon effect strongly dominates, while in Fig. 4**e**,**f** the magnetic signatures can dominate. It suggests that the comparative measurement of both magnetic domains allows extracting information on the orbital momenta of the initial states even though they are nearly quenched.

SV. MAGNETIC ANISOTROPY IN $DYMN_6SN_6$

The question of whether *easy cone* represents the physical arrangement of **M** at low temperatures in R166 compounds is currently under debate. We have recently reported that in another Kagome compound DyV_6Sn_6 the moment is canted from the c-axis by approximately 20° and projected in-plane along ΓM, however, the in-plane anisotropy between ΓM and ΓK projection is very small [7]. The older paper of Malaman et al. [8] does not really discuss the issue of in-plane anisotropy, it is possible that in these measurements it was difficult to distinguish between *easy cone* and *easy axis*. Our measurements have been performed at 20K, where small in-plane anisotropies likely fix the magnetization direction.

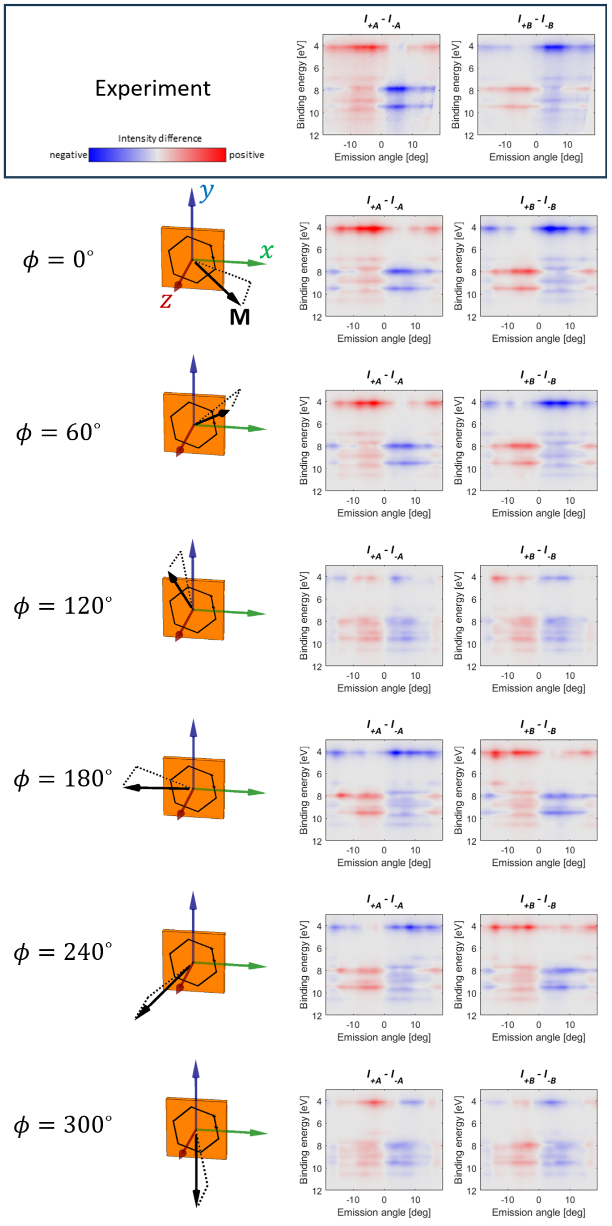

FIG. S6. Comparison between the experimental data (Fig. 2**b,c** of the main text) and calculations for various azimuthal orientations of **M** angle ϕ (polar angle kept at $\theta = 45°$). $phi = 0$ case is plotted in Fig. 2**f,g**. Black arrows in the schematic geometry images correspond to **M** in the domain A. In domain B the orientation is assumed to be -**M**.

Furthermore, our preliminary ab-initio magnetic anisotropy calculations indicate that the projection of **M** onto ΓK is energetically preferred.

Figure S5 shows our photoelectron diffraction simulation for the "*easy cone*", arrangement (we sum 12 different azimuthal projections, assuming $\theta = 45°$ polar angle). It is evident that

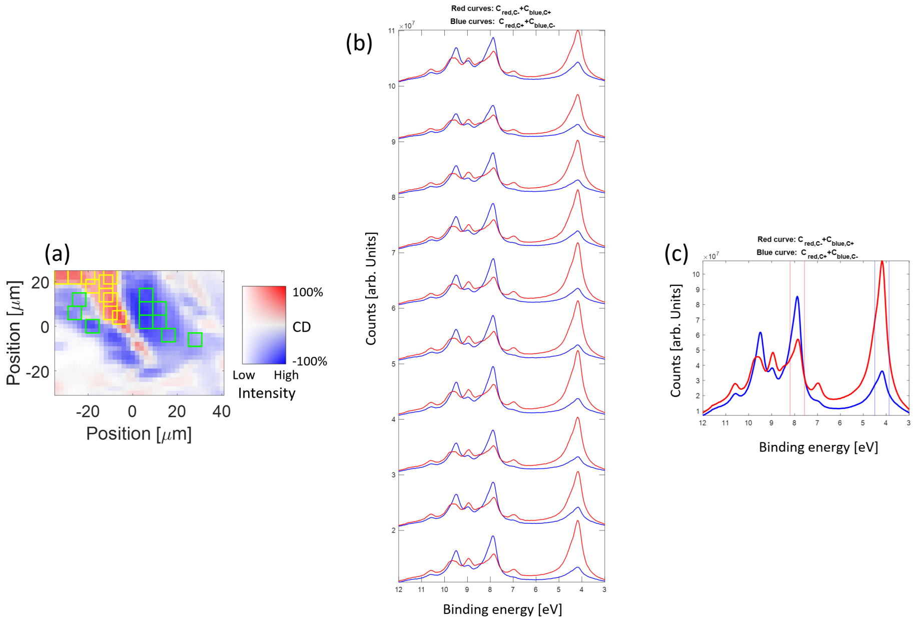

FIG. S7. Confirmation of the generality of our results, the region of Dy $4f$ at $h\nu = 300$ eV. (a) x-y micrograph similar to main text Fig. 1**d**. (b) Curves such as the one in Fig. 2**h** of the main text, but for the pairs of rectangles depicted in (a). (c) Same as (b), but sum of all rectangles in (a). The micrograph in (a) is plotted as difference of CD signals integrated over the regions indicated by vertical lines in (c).

this simulation does not correspond to the experimental data in Fig. 2**b** and 2**h**, therefore, it is an argument for the *easy axis* and against *easy cone*, in our sample at 20K.

Furthermore, we performed photoelectron diffraction calculations for all six possible projections of $\mathbf{M}$ onto the ΓK azimuth, with the results shown in Fig. S6. Only the $\phi = 0°$ and $\phi = 60°$ simulations are consistent with the experimental data, while the other azimuthal orientations of $\mathbf{M}$ show clear disagreement. In the main text, we therefore adopt the $\phi = 0°$ configuration; however, the $\phi = 60°$ geometry would also be compatible with the experiment. It is also conceivable that domain A corresponds to $\phi = 0°$ and domain B to $\phi = 60°$, although flux-closure considerations favor antialigned domains.

Considering the above, we find it plausible, that in the experiment we are probing 2 anti-aligned easy axis domains, with the $\mathbf{M}$ being at $\approx 45°$ away from c-axis and projected along the ΓK azimuthal direction.

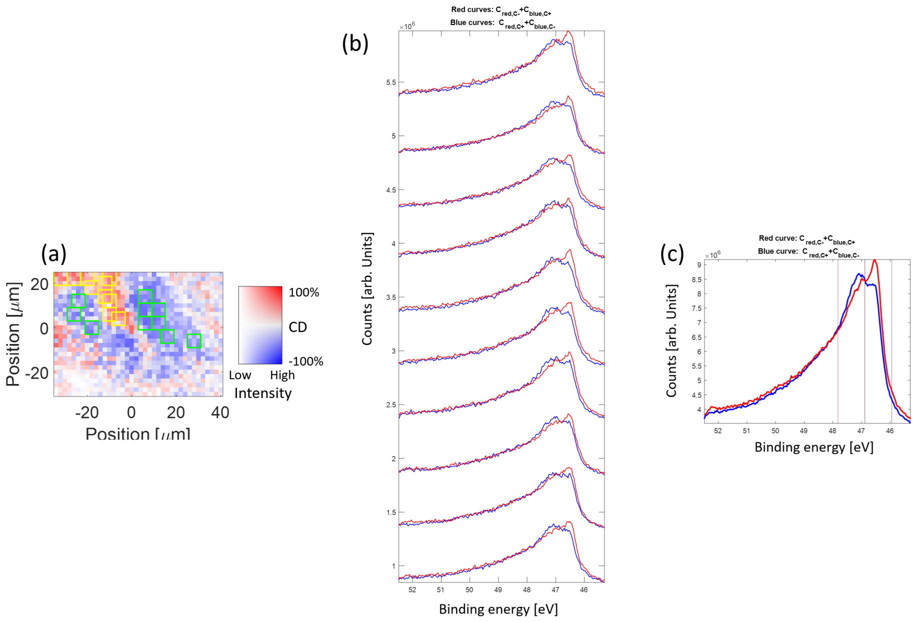

FIG. S8. Confirmation of the generality of our results, the region of Mn $3p$ at $h\nu = 300$ eV. (a) x-y micrograph similar to main text Fig. 1**f**. (b) Curves such as the one in Fig. 3**g** of the main text, but for the pairs of rectangles depicted in (a). (c) Same as (b), but sum of all rectangles in (a). Micrograph in (a) shows the CD difference between regions indicated in (c). Background correction (extracted from the low binding energy side of the spectra) has been applied in all cases.

SVI. ADDITIONAL ANALYSIS OVER LARGER SAMPLE AREA

In the main text Fig. 1 we indicated selected regions used to analyze the Dy $4f$ and Mn $3p$ spectra. In Figs. S7 and S8 we show additional analysis, where we have chosen numerous regions to demonstrate that the entire areas of the A and B domains exhibit spectra consistent with the data shown in the main text.

Furthermore, Fig. S9 demonstrates exceptional stability of the measured image over the entire course of collecting the experimental data presented in the manuscript. At MAESTRO beamline one can perform angular scans either by the electrostatic deflection system in the modified Scienta R4000 analyzer lens, or by rotating the entire electron analyzer on differential seals. During the measurements we exclusively used the electrostatic deflectors to avoid any possible beam drifts due to the weight transfer. Furthermore, the LHe cooling has been operating since several days before the measurement start, minimizing any temperature-related beam drifts.

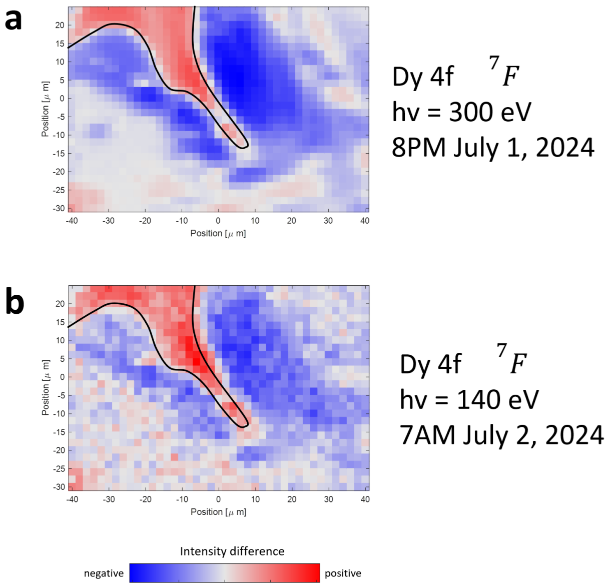

FIG. S9. Beam stability during data acquisition. Spatial maps of the Dy 4f signal acquired at the beginning (**a**) and at the end (**b**) of the experiment. The consistent spatial contrast demonstrates stable beam position and measurement conditions throughout the data collection.

SVII. CLUSTER USED IN PHOTOEMISSION CALCULATIONS

Detailed of the photoemission calculations performed using the EDAC code are explained in the Methods section of the main text. In Fig. S10 we show the visualization of the cylindrical cluster used in these calculations. From the obtained one-electron matrix elements we constructed the many-electron matrix elements (Eq. 1 of the main text) for the multiplets of Dy and Mn.

---

[1] P. Blaha, K. Schwarz, F. Tran, R. Laskowski, G. K. H. Madsen, and L. D. Marks, WIEN2k: An APW+lo program for calculating the properties of solids, The Journal of Chemical Physics **152**, 074101 (2020), https://pubs.aip.org/aip/jcp/article-pdf/doi/10.1063/1.5143061/16727313/074101_1_online.pdf.

[2] D. Wortmann, G. Michalicek, N. Baadji, M. Betzinger, G. Bihlmayer, J. Bröder, T. Burnus, J. Enkovaara, F. Freimuth, C. Friedrich, C.-R. Gerhorst, S. Granberg Cauchi, U. Grytsiuk, A. Hanke, J.-P. Hanke, M. Heide, S. Heinze, R. Hilgers, H. Janssen, D. A. Klüppel-

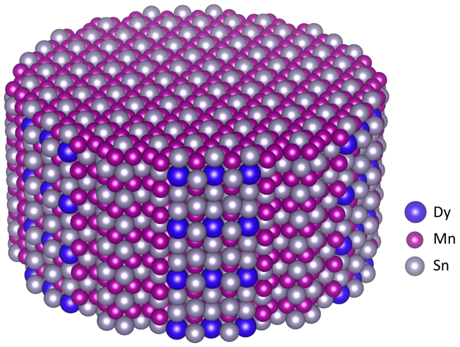

FIG. S10. Visualization of the cylindrical atomic cluster employed in the EDAC [9] multiple-scattering calculations. The cluster contains Dy, Mn, and Sn atoms arranged according to the bulk crystal structure, extending over several coordination shells to account for multiple scattering processes. This cluster is used to compute one-electron matrix elements, which are subsequently combined into many-electron matrix elements for Dy and Mn multiplets (see Methods section in the main text).

berg, R. Kovacik, P. Kurz, M. Lezaic, G. K. H. Madsen, Y. Mokrousov, A. Neukirchen, M. Redies, S. Rost, M. Schlipf, A. Schindlmayr, M. Winkelmann, and S. Blügel, FLEUR, Zenodo https://doi.org/10.5281/zenodo.7576163 (2023).

[3] H. Tan and B. Yan, Competing lattice instability and magnetism on the surface of kagome metals, Phys. Rev. B **109**, 195428 (2024).

[4] J. Yeh and I. Lindau, Atomic subshell photoionization cross sections and asymmetry parameters: $1 \leq z \leq 103$, Atomic Data and Nuclear Data Tables **32**, 1 (1985).

[5] H. Daimon, T. Nakatani, S. Imada, S. Suga, Y. Kagoshima, and T. Miyahara, Strong circular dichroism in photoelectron diffraction from nonchiral, nonmagnetic material–direct observation of rotational motion of electrons, Japanese Journal of Applied Physics **32**, L1480 (1993).

[6] S. Goldberg, C. Fadley, and S. Kono, Photoionization cross-sections for atomic orbitals with random and fixed spatial orientation, Journal of Electron Spectroscopy and Related Phenomena **21**, 285 (1981).

[7] Y. Zhou, M.-K. Lee, S. Hammouda, S. Devi, S.-I. Yano, R. Sibille, O. Zaharko, W. Schmidt, K. Schmalzl, K. Beauvois, E. Ressouche, P.-C. Chang, C.-H. Huang, L.-J. Chang, T. Brückel, and Y. Su, Ground-state magnetic structures of topological kagome metals $r\mathrm{v}_6\mathrm{sn}_6$ (r = Tb, Dy, Ho, Er), Phys. Rev. Res. **6**, 043291 (2024).

[8] B. Malaman, G. Venturini, R. Welter, J. Sanchez, P. Vulliet, and E. Ressouche, Magnetic properties of RMn_6Sn_6 (R=Gd–Er) compounds from neutron diffraction and Mössbauer measurements, Journal of Magnetism and Magnetic Materials **202**, 519 (1999).

[9] F. J. García de Abajo, M. A. Van Hove, and C. S. Fadley, Multiple scattering of electrons in solids and molecules: A cluster-model approach, Phys. Rev. B **63**, 075404 (2001).
